# Flexible intensive care unit visitation: a valuable practice that requires contextual implementation

**DOI:** 10.62675/2965-2774.20250099

**Published:** 2025-10-07

**Authors:** Cassiano Teixeira, Regis Goulart Rosa

**Affiliations:** 1 Department of Internal Medicine and Postgraduate Program in Rehabilitation Sciences Universidade Federal de Ciências da Saúde de Porto Alegre Porto Alegre RS Brazil Department of Internal Medicine and Postgraduate Program in Rehabilitation Sciences, Universidade Federal de Ciências da Saúde de Porto Alegre - Porto Alegre (RS), Brazil.; 2 Department of Internal Medicine Hospital Moinhos de Vento Porto Alegre RS Brazil Department of Internal Medicine, Hospital Moinhos de Vento - Porto Alegre (RS), Brazil.; 3 Postgraduate Program in Pneumological Sciences Universidade Federal do Rio Grande do Sul Porto Alegre RS Brazil Postgraduate Program in Pneumological Sciences, Universidade Federal do Rio Grande do Sul - Porto Alegre (RS), Brazil.; 4 Department of Critical Care Hospital de Clínicas de Porto Alegre Universidade Federal do Rio Grande do Sul Porto Alegre RS Brazil Department of Critical Care, Hospital de Clínicas de Porto Alegre, Universidade Federal do Rio Grande do Sul - Porto Alegre (RS), Brazil.; 5 Department of Critical Care Hospital Nora Teixeira Complexo Hospitalar da Santa Casa de Porto Alegre Porto Alegre RS Brazil Department of Critical Care, Hospital Nora Teixeira, Complexo Hospitalar da Santa Casa de Porto Alegre - Porto Alegre (RS), Brazil.; 6 Brazilian Research in Intensive Care Network São Paulo SP Brazil Brazilian Research in Intensive Care Network (BRICNet) - São Paulo (SP), Brazil.; 7 Department of Intensive Medicine Faculty of Medicine Universidade Federal do Rio Grande do Sul Porto Alegre RS Brazil Department of Intensive Medicine, Faculty of Medicine, Universidade Federal do Rio Grande do Sul - Porto Alegre (RS), Brazil.

While intensive care unit (ICU) visitation policies vary globally, it is widely acknowledged that most adult ICUs limit family presence at the patient’s bedside.^(1)^ These restrictions are often justified by theoretical risks tied to visitor presence, such as infections, disruptions in care, and staff burnout.^(2)^ However, the literature has not consistently confirmed these risks, and guidelines now advocate for flexible visitation as a strategy to enhance patient- and family-centered care.^(3,4)^Despite these recommendations, unrestricted ICU visiting hours remain rare. A survey by the World Federation of Societies of Intensive and Critical Care Medicine revealed that only 39% of member countries had fully embraced open visiting hours.^(5)^ Additionally, the momentum toward visitation flexibility waned during the COVID-19 pandemic.^(6)^

Flexible visitation models are increasingly proposed not only to meet patient preferences but also to improve outcomes.^(7)^ In a patient-centered context, more interaction between family members and ICU staff fosters shared decision-making, reducing exposure to modifiable risk factors for delirium, such as unnecessary sedation or benzodiazepines. However, the results regarding delirium remain controversial, with some studies indicating a reduction only when visits are extended up to 12 hours.^(8)^ Flexible hours have also been linked to lower stress levels in patients. A systematic review and meta-analysis led by Nassar et al.^(9)^ revealed reduced anxiety severity among patients in ICUs with flexible visitation. Furthermore, the review revealed no significant differences in ICU-acquired infections or mortality rates between the flexible and restricted visitation models.

Nevertheless, the term “flexible visitation” remains ambiguous. Does it mean 24-hour access? More than 12 hours? Unlimited entry and exit? An unrestricted number of visitors? This lack of a standardized definition could lead to misinterpretation and hinder comparisons across studies and implementations.^(9)^ Thus, the authors urge the establishment of a clear operational definition in future research and institutional protocols. Moreover, the structuring of flexible visitation may play a critical role in outcomes. Variables such as the total time spent at the patient’s bedside, the number of visitors, scheduled rotations, and planned communication sessions with ICU staff could influence the success of this approach.^(3)^Communication strategies, whether scheduled or unrestricted, may affect family satisfaction and staff engagement.^(3)^ In a Brazilian study exploring the perceptions of 92 family members, communication was often hindered by short interaction time with staff, inaccessible language, and inadequate locations for conversations. Family members highlighted the need for detailed information, emotional support, and participation in decision-making processes. Many also emphasized that poorly structured communication—including a lack of planning and standardization—heightened emotional distress and reduced confidence in care.^(10)^

While the real benefit of visitation flexibility appears to rest primarily with the family, families overwhelmingly prefer more open visitation policies.^(3,8)^A recent clinical trial involving over 1,200 close family members revealed that flexible visitation—combined with educational support—boosted satisfaction across key care domains: proximity to the patient, communication (how often and effectively information is shared), reassurance, emotional support, and comfort.^(8)^ Notably, the data also revealed reduced anxiety and depression among family members during ICU stays under flexible visitation, with effects persisting for up to 12 months post-ICU.^(11)^Family members felt more involved in the care of the patient, from emotional support to helping staff understand patient needs,^(12)^underscoring the value of this model in fostering both patient- and family-centered care.^(3,7)^ However, one must also consider the psychological burden imposed on some families by the implicit obligation to remain constantly at the bedside, which may induce stress or guilt.

Hospital accreditation programs are designed to ensure and improve care quality and safety.^(13)^ Accreditation evaluates health institutions on the basis of external standards of care^(14)^and often includes public reports, inspections, and performance-based incentives to promote ongoing improvements in care quality. Accreditation by organizations such as the Joint Commission or the Magnet Recognition Program often requires adherence to strict quality and safety standards, which can include specific guidelines for visitation policies.^(13)^Herein lies the problem. When accreditation demands visitation flexibility as a quality indicator, institutions may rush into compliance without proper preparation. Properly executed, flexible visitation requires comprehensive staff training (e.g., communication techniques), sufficient personnel, and psychological support within the unit. It also calls for educating families with respect to the unique environment of the ICU while ensuring that logistical considerations such as secure entry points are in place. In addition to logistical concerns, architectural and structural barriers must also be addressed. Facilities lacking designated spaces for rest, decompression, or basic amenities such as restrooms may inadvertently discourage family presence. These infrastructural limitations should be acknowledged and reflected in visual representations or figures to provide a more comprehensive view of the challenges.

Additionally, poorly planned visitation flexibility may backfire, resulting in anxious or overwhelmed family members, who, without proper guidance, could exhibit inappropriate behaviors, leading to staff dissatisfaction. This, in turn, may cause healthcare professionals to disengage from bedside care, lower their tolerance for family presence, and eventually experience job dissatisfaction and burnout.^(2,8)^ In a large national survey conducted in Brazil with 903 ICU professionals, nearly half (49%) reported that limited family visitation contributed to their burnout. Furthermore, 78% believed that restricting visits had a negative effect on patient care. Despite these perceptions, multivariable analyses revealed that visitation policies themselves were not independently associated with burnout or psychological distress; rather, financial concerns and poor communication with supervisors were the strongest predictors of staff burnout.^(15)^ Indeed, burnout often represents the final consequence of a poorly executed flexible visitation policy, turning what could be a beneficial practice into an administrative error with far-reaching implications. A large clinical trial involving more than 800 ICU staff members revealed that flexible visiting hours did not significantly affect perceptions of disorganized care or increase conflicts with visitors when an educational strategy was in place to help visitors better understand the ICU environment.^(8)^ This emphasizes the importance of clinician-centered strategies—workload reduction, communication training, and education for both families and staff—when implementing visitation flexibility to avoid staff burnout and maintain patient safety.^(3,7)^Another rarely discussed but increasingly relevant concern is staff safety. In some regions, the increase in societal violence and the threat of intergroup conflict can extend into healthcare facilities. In such contexts, changes in visitation policies must be accompanied by robust hospital safety protocols.

Ultimately, a flexible visitation policy, when appropriately executed, respects and preserves the crucial bond between critically ill patients and their families. Although implementing such a policy is complex, with many interacting components, it is both achievable and beneficial for all those involved.

## References

[1] Berwick DM, Kotagal M (2004). Restricted visiting hours in ICUs: time to change. JAMA.

[2] Giannini A, Garrouste-Orgeas M, Latour JM (2014). What's new in ICU visiting policies: can we continue to keep the doors closed?. Intensive Care Med.

[3] Davidson JE, Aslakson RA, Long AC, Puntillo KA, Kross EK, Hart J (2017). Guidelines for Family-Centered Care in the Neonatal, Pediatric, and Adult ICU. Crit Care Med.

[4] (2016). Family visitation in the adult intensive care unit. Crit Care Nurse.

[5] Kleinpell R, Heyland DK, Lipman J, Sprung CL, Levy M, Mer M, Council of the World Federation of Societies of Intensive and Critical Care Medicine (2018). Patient and family engagement in the ICU: report from the task force of the World Federation of Societies of Intensive and Critical Care Medicine. J Crit Care.

[6] Marmo S, Milner KA (2023). From open to closed: COVID-19 restrictions on previously unrestricted visitation policies in adult intensive care units. Am J Crit Care.

[7] Secunda KE, Kruser JM (2022). Patient-centered and family-centered care in the intensive care unit. Clin Chest Med.

[8] Rosa RG, Falavigna M, da Silva DB, Sganzerla D, Santos MM, Kochhann R, ICU Visits Study Group Investigators and the Brazilian Research in Intensive Care Network (BRICNet) (2019). Effect of flexible family visitation on delirium among patients in the intensive care unit: the ICU visits randomized clinical trial. JAMA.

[9] Nassar AP, Besen BA, Robinson CC, Falavigna M, Teixeira C, Rosa RG (2018). Flexible versus restrictive visiting policies in ICUs: a systematic review and meta-analysis. Crit Care Med.

[10] Cezar AG, Castanhel FD, Grosseman S (2023). Needs of family members of patients in intensive care and their perception of medical communication. Crit Care Sci.

[11] de Souza JM, Miozzo AP, da Rosa Minho dos Santos R, Mocellin D, Rech GS, Trott G (2024). Long-term effects of flexible visitation in the intensive care unit on family members' mental health: 12-month results from a randomized clinical trial. Intensive Care Med.

[12] Rosa RG, Pellegrini JA, Moraes RB, Prieb RG, Sganzerla D, Schneider D (2021). Mechanism of a flexible ICU visiting policy for anxiety symptoms among family members in Brazil: a path mediation analysis in a cluster-randomized clinical trial. Crit Care Med.

[13] Lewis K, Hinchcliff R (2023). Hospital accreditation: an umbrella review. Int J Qual Health Care.

[14] Van Wilder A, Bruyneel L, De Ridder D, Seys D, Brouwers J, Claessens F (2021). Is a hospital quality policy based on a triad of accreditation, public reporting and inspection evidence-based? A narrative review. Int J Qual Health Care.

[15] Sharma M, Wahlster S, Town JA, Patel PV, Jannotta GE, Amorim E (2024). Perceptions and preferences about family visitation restrictions and psychological distress among critical care clinicians in Brazil: results from a national survey. Crit Care Sci.

